# Brief report: Relationships of frailty and age with nonresponse to patient reported outcome measures

**DOI:** 10.1016/j.apro.2025.100197

**Published:** 2025-09-08

**Authors:** Claire R. Morton, Zara R. Cooper, Ronald Bleday, Jennifer E. Fanning, Jill Steinberg, Chengbo Zeng, Andrea L. Pusic, Jason B. Liu

**Affiliations:** a Center for Surgery and Public Health, Brigham and Women’s Hospital, United States; b Patient-Reported Outcomes, Value, and Experience (PROVE) Center, Brigham and Women’s Hospital, Boston, MA, United States; c Department of Surgery, Yale New Haven Hospital, United States; d Harvard Medical School, Boston, MA, United States; e Department of Surgery, Brigham and Women’s Hospital, Boston, MA, United States

**Keywords:** Older adults, Frailty, PROMs, Response rates

## Abstract

Patient-reported outcome measures are increasingly being used to evaluate health care interventions and systems, including in surgery. Simultaneously, older adults comprise a greater proportion of patients undergoing surgery. Frailty, a clinical syndrome more prevalent among older adults has not been examined as a factor associated with PROM response. In this single center retrospective analysis, we sought to understand the association between age and frailty with response to PROMs among a pilot group of patients. We find that within this sample of patients, adults between 66 and 84 and those with prefrailty were more likely to respond than patients less than 65 and those who are robust, respectively.

## Introduction

Patient-reported outcome measures (PROMs) objectively quantify aspects of health for which patients are the most accurate source of information. They are gaining attention as policymakers seek to hold clinicians accountable for delivering patient-centered value in health care, particularly surgical care. However, an implementation challenge of PROMs is that they require patient participation. Unequal response by demographic group may bias aggregated results from PROMs limiting representativeness, a critical challenge for further use in performance improvement and value-based models.

Since older adults disproportionately undergo more surgeries than their younger counterparts, understanding how older adults interact with PROMs is critical to ensure effective and equitable use. Frailty, or the inability to withstand physiologic stress, is associated with disability in older adults. Aspects of disability, including hearing and vision impairment are associated with reduced participation in PROMs [1]. Frailty is often assessed on the basis of comorbidity which has also been associated with reduced PROM response [2]. Finally, frailty is more prevalent with increasing age. Prior studies draw varied conclusions about the impact of age on response, including improved [2,3] or reduced response [4,5]. Our exploratory study sought to delineate the relationship between frailty and age with PROM response among surgical patients, hypothesizing that frail older patients would be less likely to respond. Such knowledge would ensure PROMs-related activities are representative and equitable.

## Methods

This is a retrospective single institution study of data from the American College of Surgeons (ACS) National Surgical Quality Improvement Program (NSQIP) PROMs Project, details reported previously [3]. The ACS NSQIP is a voluntary program available to hospitals globally that records clinical details, operations, and 30-day surgical outcomes following standardized data definitions to fuel benchmarking and quality improvement efforts [6].

The ACS NSQIP PROMs Project, conducted between February 2020 and March 2023, assessed the feasibility of collecting PROMs within the ACS NSQIP framework. A total of 65 hospitals participated in the Project. Four Patient-Reported Outcomes Measurement Information System (PROMIS) short-form measures, including the PROMIS Global-10v1.2, the PROMIS Pain Interference 4a v1.1, PROMIS Fatigue 4a v1.0 and PROMIS Physical Function v2.0 were electronically collected.

For this exploratory study using data from one of the 65 hospitals, patients were considered respondents if they completed any of the administered PROMs. Patients received up to 5 reminders and were able to complete the PROMs up to 90 days following their surgery. This exploratory analysis was determined to be quality improvement by the MGB IRB.

Frailty was defined using the 5-factor modified frailty index (mFI) which scores patients from 0 to 5 based on the cumulative presence of a fully or partially dependent functional status, diabetes requiring medication, chronic obstructive pulmonary disease, congestive heart failure, or hypertension requiring medication [7]. The mFI was selected based on available data. Patients with mFI scores of 0 were deemed “robust,” 1–2 “prefrail,” and ≥ 3 ”frail.”

Using R 4.5.0 with alpha set at 0.05, univariate tests compared demographic and clinical variables among nonrespondents and respondents. Logistic regression explored associations of frailty with response in unadjusted and adjusted analyses. Multivariable analyses assessed odds of response based on either frailty or age. When assessing response based on frailty, we adjusted for age, sex, and 30-day complications. When assessing response based on age, we adjusted for frailty, sex, and 30-day complications. Results are presented as odds ratios with 95 % CIs [8]. Odds ratios represent the odds of the outcome, in this case response to PROMs, among exposed individuals compared to the same outcome in unexposed individuals.

We conducted sensitivity analyses in which we created an interaction term of age, considered continuously or by group, with frailty to determine if the relationship between response and frailty differed by patient age. Finally, to clarify the role of functional dependence, we repeated the regression analysis with functional status as the main determinant adjusting for age, sex, and 30-day complications.

## Results

Among 3059 patients, 32.3 % participated in PROMs collection (Table 1). Response rates among robust, prefrail, and frail patients were 30 %, 35 %, and 30 %, respectively. Response rates among patients 65 and younger, between 66 and 74, 75 and 84, and 85 and older were 26 %, 41 %, 39 %, and 26 %, respectively.

Compared to respondents, nonrespondents were more frequently robust (51 % vs 46 %, *p* = 0.03) and 65 years or younger (58 % vs 43 %, *p* < 0.001) (Table 1). Black patients were more likely non-respondents than respondents (6 % vs 4 %, p = 0.01) while White patients were more likely respondents (89 % vs 85 %). Patients identified as Hispanic were more likely non-respondents than respondents (5 % vs 3 %, p = 0.005). There were no differences by functional status, sex, or 30-day postoperative complications.

On univariable regression analysis, prefrailty was associated with greater odds of response (OR 1.23, 95 % CI 1.05–1.43, p < 0.01); however, this relationship was no longer significant after adjustment for age, sex, and 30-day complications (OR 1.04, 95 % CI 0.88–1.22).

Patients aged 66–74 and 75–84 years had higher odds of response (OR 1.95, 95 % CI 1.65–2.31, p < 0.001 and OR 1.85, 95 % CI 1.47–2.33, p < 0.001, respectively) when compared to those aged 65 years or younger (Table 2). These results remained significant after adjustment for frailty, sex, and 30-day complications.

On sensitivity analyses, the interaction term of age with frailty was not statistically significant on univariable or adjusted analysis. With regards to functional status, few patients were not independent in this sample (Table 1). Accordingly, regression analysis did not reveal any statistically significant associations (Table 2).

## Discussion

In this retrospective analysis of PROMs data from a single institution, we aimed to describe the relationship between age and frailty with response to PROMs. We found that prefrailty, as defined by the mFI, showed a modest positive association with response to PROMs as did age greater than 65 years. Furthermore, race and Hispanic ethnicity, but not functional status, sex, or 30-day complications, were associated with response.

In this study, prefrailty was associated with increased response to PROMs. Though the association of frailty has not been directly examined with PROM response previously, this finding ran counter to our hypothesis based on the association of functional impairment and pre-operative comorbidities with non-response [1,2]. The relationship of prefrailty with response was not significant when accounting for age, suggesting that age was a significant confounder. The strong positive relationship between age and response rates seen in our study supports prior findings that older adults are more likely to respond to PROMs [2,3]. Our findings also align with the literature reporting increased response among non-Hispanic White patients but not with prior findings of increased responses by women [2,3,9]. These variations by racial and ethnic group, though not the focus of our study, are key considerations for implementation of PROMs in performance benchmarking in value-based healthcare.

Our study is limited in that our sample was made up of single center retrospective data. As an exploratory study we believe that these findings still contribute to an early understanding of factors associated with response to PROMs, particularly among the older adult population. Furthermore, while the mFI-5 is conveniently applied to NSQIP data, it is a limited measure of frailty. For example, patients with hypertension requiring medications would be considered prefrail under the mFI-5; however, hypertension alone would be unlikely to limit an individual’s ability to participate in PROMs. Misclassification of patients who may not meet clinical assessments of frailty, but qualify based on the mFI may have biased our results to suggest that no relationship exists. We attempted to account for this by conducting additional analyses of functional status, though this was limited by the small number of dependent patients in the sample. Future studies using more rigorous assessments of frailty are needed. Moreover, patients within our sample were predominantly White and few patients were frail, 85 years of age or older, or partially dependent. Our study was therefore likely underpowered. Despite these limitations, this pilot investigation supports the premise that frail patients, older patients, or those who may be partially dependent, are appropriate for PROMs collection.

Our results are reassuring against the risk of prefrailty compromising patients’ ability to engage with PROMs, though further work beyond this study is needed. Similarly, we demonstrate an association between age and response to PROMs in the 66–84 year old cohort that was not seen among patients 85 and over, adding nuance to prior results on the impact of age on response. These patients may be unique in that they may be retired, thus having more time to engage with PROMs, while remaining healthy enough that such efforts are not unduly burdensome. Alternatively, these patients may be most engaged with health behaviors, perceiving a time horizon for benefit that older individuals may not. Future work among patient populations with higher proportions of frailty, using additional measures of frailty, may better clarify the relationship. Additionally, qualitative work directly examining barriers and facilitators to PROMs completion among elders may identify ways to support engagement among those over 85. Other key considerations, such as incorporating proxy and caregiver responses, should also be investigated. Ultimately, our work supports the notion that older adults, including those who are prefrail, can participate in PROMs collection, underscoring the importance of PROMs for evaluating healthcare in a representative manner.

## Figures and Tables

**Table 1:** Characteristics of nonrespondents and respondents.

	Nonrespondents N = 2071 (68 %)	Respondents N = 988 (32 %)	*p*	Cramér’s V
Frailty^a^, n (%)			0.03	0.05
Robust	1049 (51)	452 (46)		
Prefrail	985 (48)	520 (53)		
Frail	37 (2)	16 (2)		
Age, y, n (%)			< 0.001	0.15
≤ 65	1208 (58)	422 (43)		
66–74	578 (30)	394 (40)		
75–84	244 (12)	158 (16)		
≥ 85	41 (2)	14 (1)		
Functional status, n (%)			0.42	0.01
Independent	2049 (99)	981 (99)		
Partially dependent	22 (1)	7 (1)		
Dependent	0 (0.0)	0 (0.0)		
Female sex, n (%)	1047 (51)	525 (53)	0.19	0.02
Race, n (%)			0.01	0.06
Asian	51 (3)	25 (3)		
Black or African American	122 (6)	36 (4)		
Other	133 (6)	48 (5)		
White	1765 (85)	879 (89)		
Hispanic ethnicity, n (%)	104 (5)	28 (3)	0.005	0.05
30-day complication^b^, n (%)	196 (10)	87 (9)	0.59	0.01
Morbidity	149 (7)	63 (6)	0.45	0.01
Unplanned reoperation	31 (2)	20 (2)	0.30	0.02
Unplanned readmission	103 (5)	48 (5)	0.93	0.00

a Determined based on the mFI-5 score. “Robust” mFI-5 score 0; “prefrail” 1–2; “frail” ≥ 3.

b 30-day complications were defined according the ACS NSQIP.

**Table 2:** Regression analysis examining associations of age and frailty with response to PROMs.

	Univariate Odds Ratio^c^ (95 % CI)	Multivariable Odds Ratio^c^ (95 % CI)
Frailty^a^		
Robust	Ref	Ref
Prefrail	1.23 (1.05–1.43)	1.04 (0.88–1.22)
Frail	1.00 (0.54–1.79)	0.79 (0.42–1.43)
Age, y^b^		
≤ 65	Ref	Ref
66–74	1.95 (1.65–2.31)	1.95 (1.64–2.32)
75–84	1.85 (1.47–2.33)	1.84 (1.45–2.33)
≥ 85	0.98 (0.51–1.77)	0.97 (0.50–1.76)
Functional status^b^		
Independent	Ref	Ref
Partially dependent	0.66 (0.26–1.49)	0.60 (0.22–1.33)

a Multivariable models adjust for age, sex and 30-day complications.

b Multivariable models adjust for frailty, sex and 30-day complications.

c Odds Ratio > 1.0 represents greater odds of response.
